# Applicability of FTIR-ATR Method to Measure Carbonyls in Blood Plasma after Physical and Mental Stress

**DOI:** 10.1155/2019/2181370

**Published:** 2019-03-26

**Authors:** Jolanta Bujok, Marlena Gąsior-Głogowska, Michał Marszałek, Natalia Trochanowska-Pauk, František Zigo, Alexander Pavľak, Małgorzata Komorowska, Tomasz Walski

**Affiliations:** ^1^Department of Animal Physiology and Biostructure, Faculty of Veterinary Medicine, Wrocław University of Environmental and Life Sciences, Norwida 31, 50-375 Wrocław, Poland; ^2^Department of Biomedical Engineering, Faculty of Fundamental Problems of Technology, Wrocław University of Science and Technology, Wybrzeże Wyspiańskiego 27, 50-370 Wrocław, Poland; ^3^Faculty of Chemistry, Wrocław University of Science and Technology, Wybrzeże Wyspiańskiego 27, 50-370 Wrocław, Poland; ^4^Department of Animal Husbandry, University of Veterinary Medicine and Pharmacy in Košice, Komenskeho 73, 041 81 Košice, Slovakia; ^5^Centre for Research in Medical Devices (CÚRAM), National University of Ireland Galway, Biosciences Research Building 118 Corrib Village, Newcastle, Galway, Ireland

## Abstract

**Introduction:**

Oxidative stress is a state of imbalance between the production of reactive oxygen species and antioxidant defenses. It results in the oxidation of all cellular elements and, to a large extent, proteins, causing inter alia the formation of carbonyl groups in their structures. The study focused on assessment of changes in the plasma protein-bound carbonyls in police horses after combat training and after rest and the applicability of infrared spectroscopy with a Fourier transform, utilizing the attenuated total reflectance (FTIR-ATR) in detecting plasma protein oxidation.

**Methods:**

We evaluated the influence of both the different concentrations of hydrogen peroxide and combat training on protein carbonylation in horse blood plasma. The oxidation of plasma proteins was assessed using a spectrophotometric method based on the carbonyl groups derivatization with 2,4-dinitrophenylhydrazine (DNPH). The measured values were correlated with the carbonyl groups concentrations determined by means of the FTIR-ATR method.

**Results:**

The linear correlation between the DNPH and FTIR-ATR methods was shown. The concentration of plasma protein-bound carbonyls significantly deceased in police horses after one-day rest when compared to the values measured directly after the combat training (a drop by 23%, p<0.05 and 29%, p<0.01 measured by DNPH and FTIR-ATR methods, respectively). These results were consistent with the proteins phosphorylation analysis.

**Conclusion:**

The FTIR-ATR method may be applied to measure the level of plasma proteins peroxidation.

## 1. Introduction

Normal cellular metabolism induces the generation of reactive oxygen species (ROS) which act as initiators of physiological redox-sensitive signaling pathways and regulators of expression of antioxidant enzymes, playing a role in the regulation of immune system status and apoptosis [1, 2]. The imbalance between ROS production and the capacity of the endogenous antioxidant defense system is defined as the oxidative stress. Oxidative stress results from exposure to physicochemical, environmental, or pathological factors; may lead to the oxidative damage of all cellular macromolecules, mainly proteins, lipids, and nucleic acids; and is widely accepted as a factor involved in the pathogenesis of many diseases such as cardiovascular disease and atherosclerosis, diabetes, or cancer [3, 4]. Although regular exercise was demonstrated to decrease morbidity in mammals, a single session of exercise results in overproduction of ROS and nitrogen reactive species, and may induce oxidative damage to lipids and proteins [5, 6]. The relationship between exercise, ROS production, and health is complex. On the one hand excessive ROS production may be involved in exercise-induced muscle damage due to oxidation of contractile proteins; on the other hand, ROS upregulate antioxidant enzymes, thus improving the antioxidant defense system function in regularly exercising individuals [7]. Moreover, psychological stress may be a factor causing oxidative stress, and if it is present during exercise, it can affect the course of oxidation processes and may have an impact on the antioxidative defense of the organism [8, 9].

The way to determine the oxidative damage caused by various physiopathological conditions is to measure the concentration of oxidation products in body fluids and tissues. Proteins are the targets of ROS, particularly because they are the most abundant components of biological systems. Oxidative modifications of proteins result mostly in polypeptide chains cleavage or cross-linking and, consequently, in loss or altering protein function and accumulation of oxidized protein aggregates. Oxidation of proteins includes multiple reactions involving both the main peptide chain and individual amino acids and is initiated by oxygen, alkyl, and nitrogen radicals [10].

The carbonyl groups on protein side chains are formed in different paths. ROS can act on proteins directly (mainly on proline, arginine, lysine, and threonine). Moreover, carbonyl groups may be introduced into proteins by the products of lipid peroxidation (malondialdehyde, 4-hydroxy-2-nonenal) or by carbonyl derivatives generated by the reducing sugars or their oxidation products [10]. Once produced, the carbonyl groups are chemically stable, which makes them easily detectable. They are present in most proteins at physiologically low levels, about 1 nmol/mg protein, which gives an average of 1 group per 3000 amino acid residues. However, during oxidative stress, their concentration increases from 2 to 8 times, which reflects well the progress of many diseases [11]. It has also been shown that their level increases with age. This makes carbonyl groups a widely used marker for oxidative modification of proteins.

**Figure 1:**
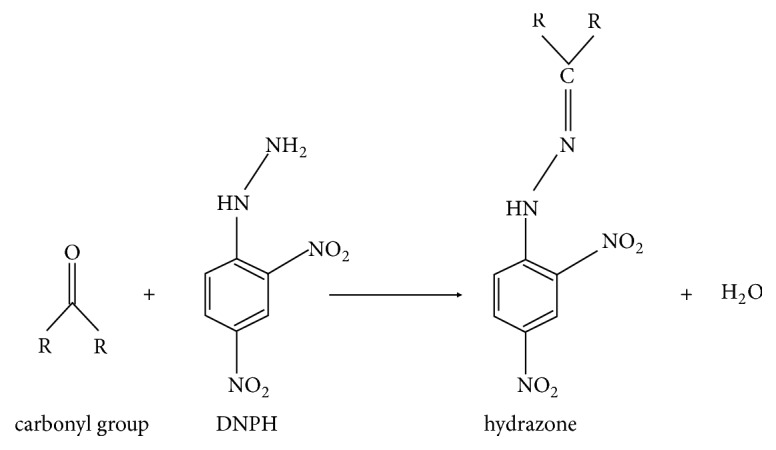
The mechanism for the reaction between the carbonyl group and 2,4-dinitrophenylhydrazine.

The standard carbonyl assay is based on the measurement of UV-VIS absorption by derivatives of these groups. The most popular is the spectrophotometric method using 2,4-dinitrophenylhydrazine (DNPH) first described by Levine [12]. The carbonyl groups in the sample react with DNPH to give a stable 2,4-dinitrophenylhydrazone product, according to the reaction shown in Figure 1. Its amount is assessed spectrophotometrically in maximum absorbance at a wavelength of 366 nm [10].

Infrared (IR) spectroscopy is one of the tools used in protein structure analyses. This method uses the fact that the molecules specifically absorb IR radiation depending on the presence of specific functional groups in them. The absorption of IR radiation excites the vibrational transitions of molecules. The energy of these transitions and, thereby, the frequency of the bond's natural vibrations correspond to the frequency of absorbed light. The theoretical background of infrared spectroscopy is described in detail elsewhere [13–15]. The ability to confirm the presence of functional groups is one of the most important advantages of this spectroscopic technique. Rapid analysis, minimal sample preparation, and the ability to measure various states of matter are the other benefits. However, water shows strong absorption in the infrared region. For this reason, many experiments are carried out on dried samples. One of the alternative solutions is using ATR equipment [16–18]. Fourier transform infrared spectroscopy with attenuated total reflectance (FTIR-ATR) has been harnessed for a wide variety of studies of biological specimens. Recent applications include whole blood analysis [19–21] as well as its fractions [22–24], protein [25, 26], or lipid components studies [27]. ATR-FTIR has also proved useful in the oxidative stress monitoring of different blood components in various conditions, e.g., during extracorporeal circulation or chronic psychological stress [8, 23, 27]. Moreover, FTIR was used to evaluate the effect of chronic psychological stress and piracetam on oxidative changes in mononuclear cells in rats [9].

The aim of our experiments was to test the usefulness of FTIR-ATR technique in the assessment of the protein oxidation in blood plasma of exercising police horses. Results obtained from FTIR-ATR spectra analysis were compared with spectrophotometric carbonyl assay utilizing DNPH.

## 2. Materials and Methods

### 2.1. Blood Plasma Preparation

The blood was collected from 10 horses (three mares and seven geldings, aged 6-20 years, mean 9.6 ± 4.2 years) owned by Police Force of the Slovak Republic for performance evaluation. Blood was collected by a jugular vein puncture immediately after the training and after 16-hour rest. The training lasted 6 hours and consisted of alternating low-intensity exercises: walking, trotting, jumping, and galloping over short distances under mental stress conditions (water cannons, dogs, simulated aggressive crowd). After the training horses were kept overnight in individual boxes in the stable. Samples were immediately transported in a cooled container to the laboratory of the University of Veterinary Medicine and Pharmacy in Košice for routine diagnostics. The plasma for protein oxidation assays was obtained by centrifugation of the remaining heparinized blood (5000 RPM for 10 minutes). The material was collected into 2 Eppendorf tubes and immediately placed at -80°C. Afterward, the plasma was transported in thermally insulated containers to the laboratory of the Department of Biomedical Engineering in Wrocław for protein carbonylation assays.

Blood samples after training as well as those after rest were processed for DNPH method and FTIR-ATR.

### 2.2. Oxidation of Proteins in a Suspension Using Hydrogen Peroxide

The experiment was conducted based on the previously described method [27], in which lipid peroxidation was measured. The test consisted of subjecting the plasma sample to the oxidation using hydrogen peroxide (H_2_O_2_; Sigma-Aldrich, USA). A series of 7 dilutions of 30% (9.8 M) H_2_O_2_: 1, 2, 5, 10, 20, 50, 100 mM was made. Samples were incubated for 30 minutes at 37°C in a thermomixer (HLC BioTech MHR 11). Then, each sample was divided into two parts. The first part was used to determine the concentration of protein carbonyls using a spectrophotometric method (DNPH) with absorbance at 366 nm. The second part was intended for testing by means of the FTIR-ATR spectroscopy. The experiment was repeated three times for both methods (n = 3).

### 2.3. Spectrophotometric Determination of Carbonyl Groups Using 2,4-Dinitrophenylhydrazine

Plasma nucleic acids were precipitated with 1% streptomycin sulfate (stock solution, 10%, in 50 mM HEPES, pH 7.2, Sigma-Aldrich, USA). Samples were allowed to stand for 15 min. Afterward, they were centrifuged at 8000 g for 10 minutes at 4°C. 25 *μ*l of such a prepared sample was added to 1 control and 3 test tubes. 500 *μ*l of the HCl solution and 500 *μ*l of the DNPH solution (10 mM in 2 M HCl, Acros Organics, Belgium) were pipetted into the control and test tubes, respectively. Samples were left for 1 hour in the dark at room temperature and stirred every 10 minutes. After this time, 500 *μ*L of a 30% trichloroacetic acid solution (Chempur, Poland) was added to each tube, mixed, and incubated at 4°C for 10 min followed by centrifugation at 11000 g for 15 minutes at 4°C. The supernatant was discarded, and 1 ml of ethanol/ethyl acetate (Stanlab, Poland, and Sigma-Aldrich, USA, respectively) (1:1, v/v) was added to the protein precipitate. Then, it was mixed thoroughly, incubated for 5 minutes, and centrifuged at 14000 g for a further 5 minutes. This step was repeated three times. Subsequently, 500 *μ*l of 6 M guanidine chloride (GdmCl; Sigma-Aldrich, Germany) dissolved in 2 M HCl was added to the collected proteins and stirred in a thermomixer at 37°C for 15 minutes (until the entire precipitate dissolved). The absorbance was measured in a spectrophotometer at the wavelength range of 360-370 nm using UV cuvettes (BRAND GMBH + CO KG, Germany). Protein carbonyl content in horse plasma was normalized to total protein concentration measured by colorimetric Biuret assay in crude plasma samples using a commercial reagent (Alpha Diagnostics Sp. z o.o., Poland) [10, 28].

### 2.4. A Test Using FTIR Spectroscopy

All FTIR-ATR spectra were collected using a NICOLET 6700 spectrometer (Thermo Scientific, USA) equipped with ATR Accessory with Heated Diamond Top-plate (PIKE Technologies, USA). The spectrometer was constantly purged with dry and carbon dioxide-free air. Plasma sample volumes of 20 *μ*L were pipetted onto the ATR crystal. To reduce the moisture in the sample, the vacuum pump was used. Spectra were recorded with a resolution of 4 cm^−1^ with 128 coadded scans over the range 4000-700 cm^−1^ at a constant temperature of 25°C. Each sample was recorded three times, and the average spectrum was calculated.

Data analysis was performed using OMNIC 8 and Origin Pro 8.5 software. Spectra were ATR and baseline-corrected [16, 29], smoothed with Savitzky–Golay [30] polynomial filter (polynomial order 2, window with 31 points) and normalized to 1 for the Amide II band at 1549 cm^−1^.

### 2.5. Statistical Analysis

The obtained results were analyzed using the statistical package Statistica 12.5, produced by StatSoft. The Kolmogorov–Smirnov, and Shapiro–Wilk tests were used to investigate the normality of the distribution of data. Linear regression was used to examine the relationship between the two examined methods. The paired t-test was used to compare results obtained after the horses training and after the recovery. Differences were considered significant at p < 0.05.

## 3. Results and Discussion

### 3.1. Results of Protein Peroxidation Using H_2_O_2_

#### 3.1.1. DNPH Spectrophotometric Method

**Figure 2:**
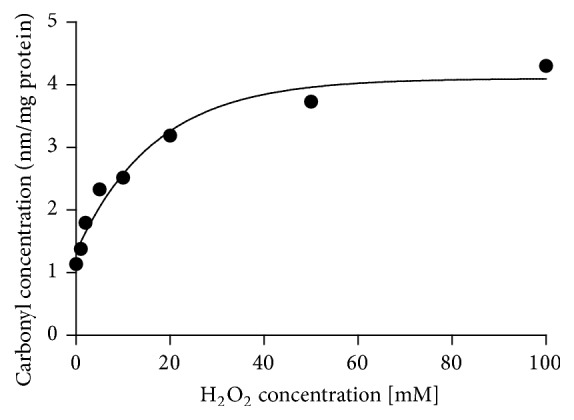
Dependence of the carbonyl content changes upon the concentrations of H_2_O_2_ measured by DNPH method.

The first test was made to induce oxidative stress in crude plasma samples using hydrogen peroxide to prove the effectiveness and repeatability of the methods that we developed and to determine the correlation between the increasing concentration of hydrogen peroxide and the level of carbonyl groups. The obtained results indicate an increase in the absorbance of the carbonyl groups in relation to the increasing concentration of hydrogen peroxide.  Figure 2 shows the trend of this increase, which is asymptotic for the entire range of hydrogen peroxide. Although spectrophotometric DNPH assay does not provide detailed information on the type of oxidized proteins, we clearly demonstrated that plasma proteins are the target of oxidative damage in horses. Albumin seems to be the most susceptible to carbonylation [21, 31]. In the plasma of mice, two proteins were distinguished, which showed an increase of carbonylation in relation to age: albumin and transferrin; in rats: albumin and *α*-macroglobulin; and in the Rhesus monkeys: also albumin and other unidentified protein. In humans, however, as a result of oxidation of plasma* in vitro*, mainly carbonylation of fibrinogen was demonstrated and, to a lesser extent, of transferrin, immunoglobulins G and A, and albumin [21].

#### 3.1.2. FTIR-ATR Method

**Figure 3:**
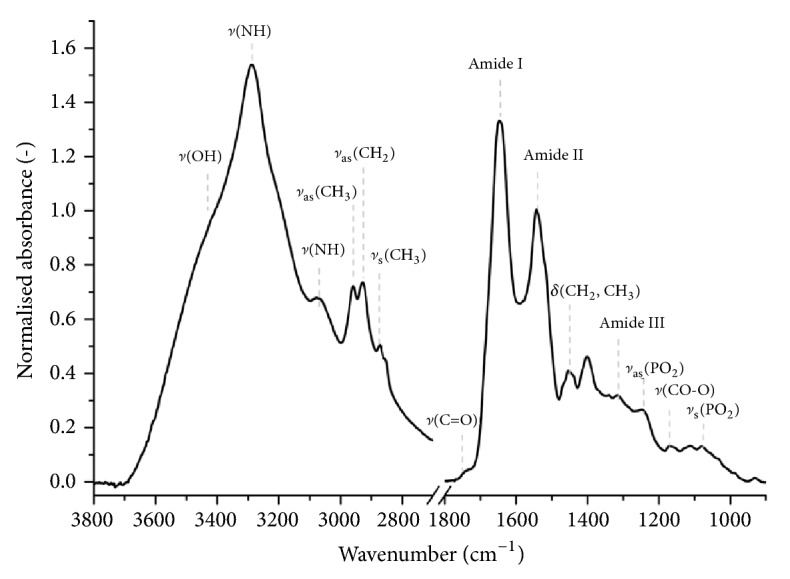
A representative FTIR-ATR spectrum of a dry plasma film.

In the FTIR-ATR spectrum of blood plasma (Figure 3) proteins predominate [32, 33], in particular, albumin [25]. The most intensive band at ~3285 cm^−1^ is assigned to NH stretching vibrations of peptide groups (Amide A). This band is superposed with the broadband arising from OH stretching vibrations of hydrogen-bonded water. The shoulder band at ~3070 cm^−1^ is also assigned to the NH stretching vibrations of proteins (Amide B). Strong bands in range 3000-2800 cm^−1^ correspond to CH stretching vibrations of the methyl and methylene groups of both protein and lipid components of blood. Fatty acids, cholesterol esters, and triglycerides produce a weak band at 1742 cm^−1^ reflecting the C=O stretch mode of carbonyl ester groups. It is a well-known marker for peroxidation of lipids [23, 34]. Two most prominent bands at 1645 and 1545 cm^−1^ are assigned to Amide I and Amide II band, respectively. The Amide I band arises mainly from C=O stretching vibrations of the peptide bond, while the Amide II is associated with the C-N stretching vibrations and N-H bending modes. Both bands are sensitive to the secondary structure of proteins [13, 14]. In turn, phosphate stretching bands at ~1244 and 1078 cm^−1^ have been found to be responsive to protein phosphorylation caused by oxidative stress [34]. More comprehensive assignment of infrared bands of blood plasma has been provided elsewhere [33, 35, 36].

**Figure 4:**
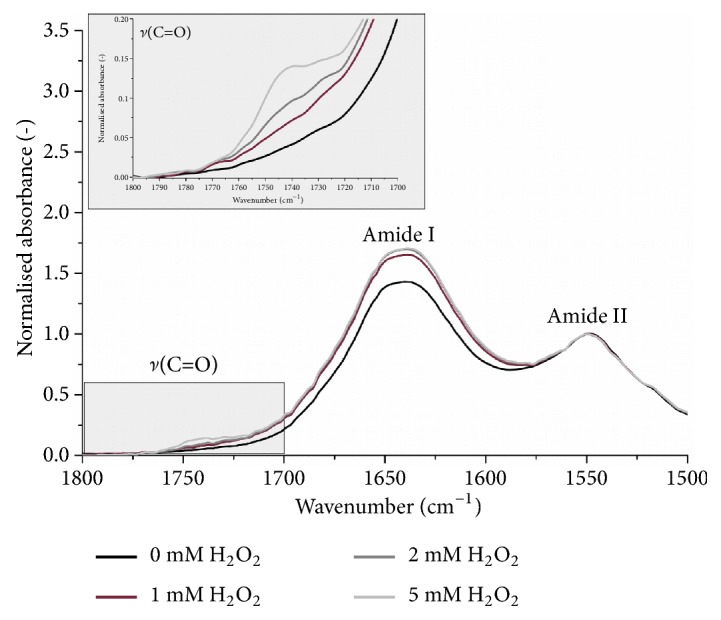
Changes in the FTIR-ATR spectrum of dry blood plasma film due to H_2_O_2_ addition: 1800-1500 cm^−1^ and *ν*(C=O) band region highlighter.

**Figure 5:**
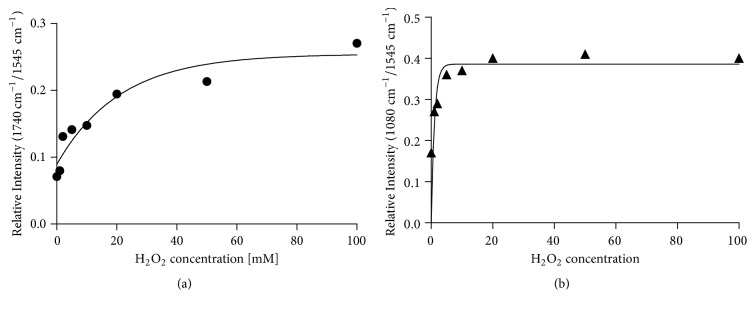
Dependence of change in the ratio of integral band absorbance (normalized *ʋ*(C=O/Amid II)) (a) and normalized *ʋ*(PO_2_^−^/Amid II) (b) upon the concentration of H_2_O_2_.

The addition of the H_2_O_2_ to the plasma causes significant distortion in its absorbance spectrum.  Figure 4 shows that changes occurred within two regions of interest: carbonyl/amide region (1800-1500 cm^−1^) dominated by protein bands and carbohydrate/phosphate region of 1125-975 cm^−1^. Increasing intensity of the carbonyl band (1740 cm^−1^) is observed, which is mainly related to the increase of aldehyde groups from lipid peroxidation [23, 27]. However, it was found that oxidation of methionine [37] can be monitored by changing the intensity of this band, as well as a band near 1040 cm^−1^. This peak is assigned to S* *=* *O stretching vibration in methionine sulfoxide [38] and to stretching C-O vibrations in carbohydrates [32, 39]. With the second band around 1080 cm^−1^, it is the marker of glucose content in blood [39]. In turn, the band at 1080 cm^−1^ is often assigned to PO^2-^ symmetric stretching of phospholipids [27] and nucleic acids [36]. Although the FTIR-ATR spectrum of blood plasma is very complex and the assignment of absorbance bands to major components of tissue is not unequivocal, our studies clearly show that this technique is sensitive to the protein peroxidation. We proved that the ratio of integrated intensity of *ν*(C=O) at 1740 cm^−1^ band to Amide II is exponentially associated with the amount of added hydrogen peroxide (Figure 5(a)).

The broadening of Amide I band is also visible, which was found to be evidence of protein oxidation [40]. Full Width at Half Maximum (FWHM) values of this peak for H_2_O_2_ treated plasma were elevated by an average of 2 cm^−1^. In addition, an exposure to the oxidizing agent causes protein aggregation that results in an increase of 1630 cm^−1^/1645 cm^−1^ ratio. In turn, the phospholipids deesterification process gave rise to phosphate bands (1240 and 1080 cm^−1^) originating from proteins. Peroxidation of phospholipids leads to protein phosphorylation and it is well-known that oxidative stress promotes that phenomena [41, 42]. We found that there is a clear correlation between added H_2_O_2_ and normalized intensity of the band at 1080 cm^−1^ in plasma FTIR-ATR spectrum (Figure 5(b)).

#### 3.1.3. Comparison of DNPH Spectrophotometric Method and FTIR-ATR Method

**Figure 6:**
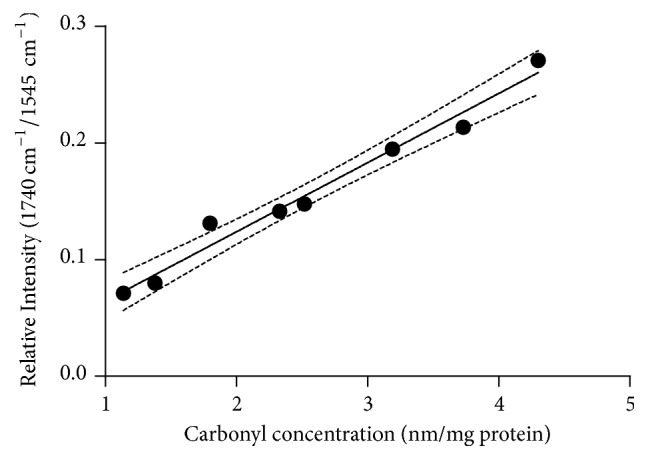
A simple linear regression of changes in the intensity of the absorbance corresponding to the peroxidation of proteins measured as the content of carbonyl groups measured by FTIR-ATR technique against changes measured by DNPH. Linear regression equation: ΔRI_FTIR_ = 0.059·ΔRI_DNPH_ + 0.005; R^2^ = 0.9764. The dashed line indicates 95% confidence intervals.

In the peroxidation study of proteins using hydrogen peroxide, both methods have shown that the carbonyl groups are being formed with increasing hydrogen peroxide concentrations.  Figure 6 shows that there is a strong, positive, linear correlation between the two mentioned methods. Comparing both methodologies, spectrophotometric DNPH assay is multistep and more labor-intensive. Therefore, the method is difficult to apply for fast screening of plasma protein oxidation when a large number of samples should be analyzed. Moreover, many steps in the spectrophotometric DNPH assay including washing, protein pelleting, and resolubilization make it more likely to produce false results [43]. On the contrary, FTIR-ATR method requires no additional sample preparation except vacuum drying making it suitable for rapid measurement of many specimens reducing water contribution to the IR spectrum.

### 3.2. Protein Peroxidation in Police Horses: In Vivo Study

**Figure 7:**
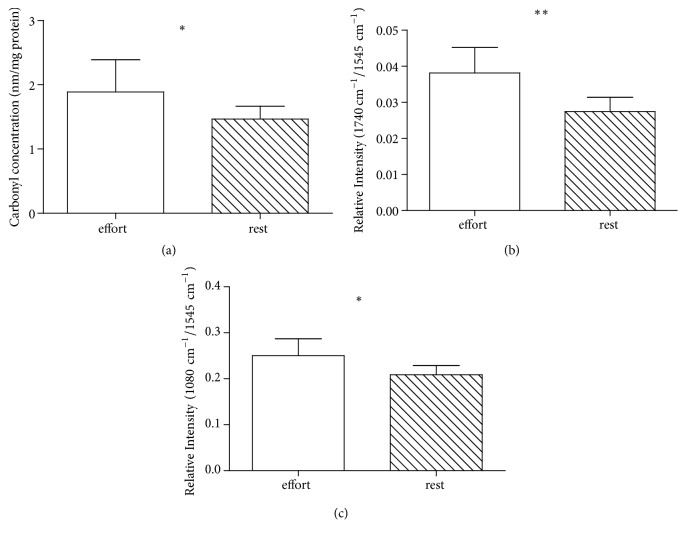
Changes in the level of carbonylation of plasma proteins in police horses after combat training and after rest measured by the DNPH method (1.89 ± 0.50 and 1.47 ± 0.19, respectively; p = 0.0178) (a) and the FTIR-ATR technique (normalized *ʋ*(C=O/Amid II)) (0.038 ± 0.007 and 0.027 ± 0.004, respectively; p = 0.0059) (b) Corresponding results of proteins phosphorylation measured by the FTIR-ATR (normalized bands ratio (1080 cm^−1^/Amid II)) (0.25 ± 0.04 and 0.21 ± 0.02, respectively; p = 0.0343) (c) Data are expressed as the mean (SD) (n = 7). *∗*p < 0.05, *∗∗*p < 0.01, effort vs rest.

In all horses, a concentration of carbonyl groups in plasma was significantly higher immediately after the training compared with the recovery period measured with DNPH spectrophotometric assay (p<0.05; Figure 7(a)) and FTIR-ATR method (p<0.01; Figure 7(b)). This suggests an increase in plasma protein carbonylation as well as protein phosphorylation (p<0.05; Figure 7(c)) due to oxidative stress in horses during low-intensity moderate-duration exercise. Skeletal muscles are considered the major source of the ROS during exercise because of increased oxygen consumption. Mitochondrial electron leak and xanthine oxidase activity contribute to superoxide anions generation. Moreover, nicotinamide adenine dinucleotide phosphate (NADPH) oxidase from activated inflammatory cells, as well as autooxidation of catecholamines and production of methemoglobin, is further source of ROS [44]. While superoxide anion generated in the mitochondria of muscle cells is considered to cause mainly actin and myosin heavy chains carbonylation within the cells, superoxide released by the enzymes on the plasma membrane and uncharged ROS are capable of oxidizing plasma proteins [45]. In line with our results, elevated concentrations of protein carbonyls were detected after exercise in skeletal muscles and blood plasma in horses after a single bout of moderate intensity exercise and after 2-day competition [46, 47]. Moreover, similarly to our results, after a moderate exercise in trotters muscle and plasma carbonyls returned to pre-exercise values at the 24^th^ hour of recovery [46]. This may be related to high antioxidant defense system capacity in animals regularly subjected to medium intensity aerobic physical activity. Opposite results were observed in people after intensive acute anaerobic training or after long-term exercise (ultramarathon). In these subjects increased plasma carbonyl concentrations were noted after 24 h and 7 days, respectively [48, 49]. It may be related to the depletion of antioxidant reserves and the intensification of ROS generation as a result of hypoxia. This is supported by the results of research on oxidative stress induced by training at high altitudes in hypoxic conditions, where the prolonged recovery period in unacclimatized individuals was observed [50].

## 4. Conclusions

During* in vitro* model studies, a correlation between the results obtained using FTIR spectroscopy and the results of the DNPH method was investigated. The results obtained by both methods are highly consistent. In trained police horses subjected to low-intensity, moderate-duration exercise, the concentration of protein oxidation products in blood plasma decreased within 16 hours of recovery. Both the DNPH assay and FTIR-ATR method yielded comparable results while FTIR-ATR was less labor-intensive and presented the lower probability of false results. In conclusion, the usefulness of the proposed method for studying plasma protein peroxidation was confirmed.

## Data Availability

The data used to support the findings of this study are included within the article.
